# Accurate Control of 17β-Estradiol Long-Term Release Increases Reliability and Reproducibility of Preclinical Animal Studies

**DOI:** 10.1007/s10911-016-9368-1

**Published:** 2016-11-26

**Authors:** Céline Gérard, Anne Gallez, Charline Dubois, Pierre Drion, Philippe Delahaut, Etienne Quertemont, Agnès Noël, Christel Pequeux

**Affiliations:** 1 Laboratory of Tumor and Development Biology, GIGA-Cancer, University of Liege, CHU-B23, Hippocrate avenue 13, B-4000 Liège, Belgium; 2 Experimental Surgery unit, GIGA & Credec, University of Liege, 4000 Liège, Belgium; 3 CER Groupe, Health Department, 6900 Marloie, Belgium; 4 Department of Psychology, Cognition and Behavior, University of Liege, 4000 Liège, Belgium

**Keywords:** Estrogen, Estradiol, Hormone administration, Matrix, Pellet, Reservoir, Implant, Pharmacokinetics, Mouse, Rat, Slow-release, Animal welfare

## Abstract

Estrogens are the subject of intensive researches aiming to elucidate their mechanism of action on the various tissues they target and especially on mammary gland and breast cancer. The use of ready-to-use slow releasing devices to administer steroids, especially estrogens, to small experimental animals remains the method of choice in terms of animal well-being and of safety for both the researcher and the animal. In this study, we evaluated and compared, in vitro and in vivo, the release kinetic of estradiol (E2) over sixty days from two different slow-releasing systems: the matrix pellet (MP) and the reservoir implant (RI). We compared the impact of these systems in three E2-sensitive mouse models : mammary gland development, human MCF7 adenocarcinoma xenograft and mouse melanoma progression. The real amount of E2 that is released from both types of devices could differ from manufacturer specifications due to inadequate release for MP and initial burst effect for RI. Compared to MP, the interindividual variability was reduced with RI thanks to a superior control of the E2 release. Depending on the dose-dependent sensitivity of the physiological or pathological readout studied, this could lead to an improvement of the statistical power of in vivo experiments and thus to a reduction of the required animal number. Altogether, our data draw attention on the importance to adequately select the slow-releasing device that is the most appropriated to a specific experiment to better fulfill the 3Rs rule (Replacement, Reduction, Refinement) related to animal welfare and protection.

## Introduction

Estrogens play a key role in sexual development and reproduction. They are also implicated in a large number of other physiological and pathological events including cancer progression [1–8]. The elucidation of the molecular mechanisms sustaining steroid action on the various tissues they target and especially on mammary gland and breast cancer is the subject of intensive researches. The development of selective estrogen receptor modulators (SERM), such as tamoxifen, has provided important improvement for the treatment of estrogen receptor (ER)-positive breast cancer [9, 10]. The use of animal models, especially rodents, is crucial for elucidating the biological effects and molecular mechanisms of 17β-estradiol (E2) and SERMs. A substantial part of the experimental animal studies have been conducted in ovariectomized rodents receiving exogenous E2, to avoid the cyclic influence of ovarian hormones [11–16]. However, there is a lack of consensus about the optimal means to reach appropriate E2 plasma concentrations and maintain long-term steady state E2 plasma level.

Estrogens are usually administered orally to humans. Several oral administration formulations have been developed for rodents and include gavage, food-mixture preparations [17, 18] or supplemented drinking water [19, 20]. These methods present several drawbacks such as stressful animal handling, difficulty of mixing hydrophobic estrogens in water, impossibility to precisely control the individual intake or induction of pulsatile concentration of E2 in plasma. The other modes of administration are daily subcutaneous injections or subcutaneous implanted slow-releasing devices [21]. These slow-release formulations have clear benefits as they are easily inserted by a single light surgery, they do not require daily stressful animal manipulation and they avoid pulsatile concentration observed with daily injections.

Based on drug release mechanisms [22], slow-releasing devices are classified in three categories: osmotic systems, matrix systems and reservoir systems.

The Alzet^®^ system is based on the osmotic pumping mechanism and consists of an osmotic compartment surrounded by a semi-permeable membrane with a single delivery orifice. In an aqueous environment, water is imbibed through the semi-permeable membrane, generates an osmotic flux and builds up a hydrostatic pressure pushing the drug solution out of the implant through the orifice. The drug release rate is generally constant as long as the osmotic driving force from the water influx is constant. The maximum release time is six weeks for small animals (www.alzet.com). Although this system is more appropriated for highly water soluble active agents, it can be used for steroids but it is necessary to solubilize them first in ethanol.

In a matrix device, the drug is dissolved or dispersed in a polymeric carrier or lipid matrix. In this system, drug molecules can elute out of the matrix by dissolution or erosion in the surrounding excipient and diffuse through the matrix structure, without any control of the release. As consequence, the drug release is not constant and decreases over time, proportionally to the square root of time. Slow-release matrix pellet are commercially available for several steroids and SERMs, in a large range of concentrations, covering 21 to 90 days of release (www.innovrsrch.com).

In the reservoir system, the drug core is surrounded by a rate-controlling membrane. Drug diffusion is driven by the drug concentration gradient across the membrane, according to the first order law of Fick. As a large excess of drug is present inside the reservoir to maintain saturation on the upstream side of the membrane, a constant rate of drug release is preserved. Administration of steroids by reservoir systems can be achieved either by newly commercially available implants (http://www.belmatech.com/en/) or by home-made silastic^®^ capsules. However, the home-made silastic^®^ capsules imply the preparation of the active agent dispersed in oil or mixed with cholesterol powder in various proportion and the filling of the silastic^®^ tube (inner diameter ± 1.5 mm) [17, 18, 23].

It is important to mention that steroid manipulation carry some health hazards. Handling of hormones in crystalline powder form or in solution must occur in a functional fume hood with appropriate protection for the staff. Risks include fertility damage and carcinogenicity, besides of clothes and environment contamination (see safety data sheet such as http://www.caymaneurope.com/msdss/10006315m.pdf). For all these reasons and because subcutaneous implantable devices must fulfill criteria like sterility and apyrogenicity, ready-to-use commercial slow-releasing devices present significant advantages in terms of safety and represent the device of choice for experimental research on rodent.

Different drug release devices could induce variations in term of drug pharmacokinetic and thus could influence the physiological endpoints being measured in an experiment. The aim of the present study was: 1) to evaluate and compare, in vitro and in vivo, the release kinetic of E2 over sixty days of two industrially manufactured implantable formulations (matrix pellet (MP) from Innovative Research of America (IRA), and reservoir implant (RI) from Belma Technologies (BT)), and 2) to investigate the impact of these systems on three E2-sensitive mouse models: mammary gland growth, human mammary tumor xenografts and mouse B16K1 melanoma.

## Materials and Methods

### Chemicals

Slow-release matrix pellets (MP) were purchased from IRA (SE-121, Sarasota, FL, USA). Reservoir implants (RI) for rat and mouse were purchased from Belma Technologies (RE2–60/ME2–60, Liège, Belgium). E2 (purity ≥98%) was purchased from Nanjing Hanchen Medical & Chemical Co., Ltd. (Nanjing, China). Deuterium labeled internal standard (IS) 17β-estradiol-2, 4, 16, 16, 17-d_5_ (d_5_-E2) was obtained from C/D/N Isotopes Inc. (Quebec, Canada). HPLC grade methanol was from J.T. Baker® (Deventer, Netherlands) and ultrapure water was dispensed from a Milli-Q purification system (Millipore Corporation, Bedford, MA, USA). Phosphate buffer (pH 7.4; 50 mM) was prepared with sodium hydroxide from VWR (Leuven, Belgium), and with potassium dihydrogen phosphate and sodium azide from Merck (Darmstadt, Germany).

### In Vitro Drug Release

MP (0.01 and 1.7 mg/60 days) and RI (ME2/60 days, ME2L/60 days and RE2/60 days) were placed in amber bottles containing 200 ml of phosphate buffer at pH 7.4 and maintained in a 37 °C water bath agitating at 140 rpm [24]. E2 concentration was determined after 1, 2, 3, 7, 10, 14, 21, 28, 35, 43, 49, 56 and 63 days. To respect sink condition, release medium was completely withdrawn and replaced with fresh buffer 24 h before each time point. Sample aliquots were then taken and E2 was quantified using LC-MS/MS technique as detailed below.

### LC–MS/MS Analysis

Chromatographic separation was achieved on a Agilent HP 1100 series (Agilent Technologies, Waldbronn, Germany) controlled by a LC Chemstation. MS detection was carried out using an Ultima triple quadrupole instrument (Micromass, Manchester, United Kingdom) operating under MassLynx 4.1 (Waters) and configured with a Z-spray electrospray ionization source operating in the negative ion mode. The isocratic separation was performed on a 150 mm × 2.1 mm Altima C18 column with 3 μm particles (Grace, Lokeren, Belgium) using a mobile phase consisting in a mixture of methanol and purified water (60/40, *v*/v). The flow rate was settled at 0.3 ml/min, the sample injection volume was of 20 μL and the column temperature was 35 °C. Multiple reaction monitoring (MRM) mode was used to monitor E2 at m/z 271/183 and 271/145, and the internal standard d5-E2 at m/z 276/187.

The e-noval software v3.0 (Arlenda S.A., Liège, Belgium) was used to validate the analytical method. Six point calibration curves were constructed and the linearity was determined by plotting the peak area ratio of E2 to d_5_-E2 versus the hormone concentration. Standard curves were generated from four independent runs. Good linearity was obtained with a weighted (1/X^2^) quadratic regression. Curves were linear from 1 to 500 ng/ml and correlation coefficients (r^2^) ranged from 0.9977 to 0.9986. The detection limit was 0.16 ng/ml. Validation experiments were made by means of four series (one series per day). Trueness expressed in term of relative bias (%) was assessed from the validation standards at seven concentration levels and ranged from −2.304 to 3.860. The precision was then determined by computing the relative standard deviation for repeatability and between-series intermediate precision at each concentration level of validation standards. Values ranged from 2.523 to 5.241 and from 3.265 to 6.267 respectively depending on the concentration level.

### Hormone Assays

Electrochemiluminescence immunoassay (ECLIA) on a Modular Analytics E analyzer (Roche, Basel, Switzerland) was used to determine plasma samples concentration of E2. According to the manufacturer, measuring range of the master curve was from 5 to 4300 pg/mL which defined the lower and the maximum detection limits. Repeatability and intermediate precision ranged from 2.4–4.6% and 4.3–9.9% respectively depending on the concentration level. The entire analysis was performed in a single run.

### Ethic Statement of in Vivo Studies

All experimental procedures and protocols used in this investigation were reviewed and approved by the Institutional Animal Care and Use Ethics Committee of the University of Liège (Belgium). The “Guide for the Care and Use of Laboratory Animals”, prepared by the Institute of Laboratory Animal Resources, National Research Council, and published by the National Academy Press, was followed carefully as well as European and local legislation. Animal welfare was assessed at least once per day with humane endpoints applied when necessary as described in the ethical form.

### In Vivo Drug Release

Ovariectomized Sprague Dawley female rats weighing approximately 300 g were purchased from the Central Animal Facility of the University of Liège -LA2610359-, Belgium. Rats were subcutaneously (s.c.) implanted with a 1.7 mg/60 days MP (*n* = 6) or with a RE2/60 days RI (*n* = 6). Blood samples were collected after 5, 12, 19, 32, 47 and 60 days by tail lateral vein puncture into heparinized Tube (Multivette®600 Tubes, Sarstedt, Essen, Belgium). Ovariectomized mice were s.c. implanted with ME2/60 days or ME2L/60 days RI. Blood were collected by cardiac puncture into serum tubes (Minicollect® Tubes, Greiner bio-one, Vilvoorde, Belgium) at 2, 7, 14, 28 and 63 days. Blood samples were centrifuged at 2,500×g for 10 min. Plasma samples were kept at −20 °C until analysis. Uterus were weighted, collected, fixed using 4% formalin and embedded in paraffin for histological analysis at each time point. Sections were cut at 6 μm, deparaffinised in xylene and rehydrated through graded alcohols, then stained with hematoxylin/eosin.

### Mammary Gland Model

C57BL/6 female prepubertal mice (4 weeks, *n* = 20) were purchased from Janvier Laboratory (Saint-Berthevin, France) and ovariectomized under isoflurane anesthesia to remove endogenous ovarian hormone production. Mice were housed under a standard 12-h photoperiod with food and water provided ad libitum. Treatments were initiated at 35 days of age for a period of 14 days. Mice (5 animals in each group) were fed by gavage with peanut oil containing 5% ethanol (vehicle, negative control) or with E2 (1 mg/kg/day), or were implanted s.c. with either a ME2/60 days RI or with a MP of E2 (0.01 mg/60 days). At the end of the treatment, mice were euthanasized and mammary tissues were dissected for whole mount preparation. One of the fourth inguinal mammary glands was removed in one piece and spread onto a glass slide. The removed gland was subjected to whole-mount fixation, defatting and staining. The mammary glands were fixed in Carnoy’s fixative (ethanol, chloroform, glacial acetic acid; 6:3:1) for at least 4 h and defatted in acetone over-night. They were then re-hydrated through a series of graded alcohols and stained with carmine overnight. Whole mount glands were then dehydrated sequentially through 70%, 90% and 100% ethanol for 20 min each and cleared in xylene for at least 2 h. Digital pictures of mammary glands immersed in xylene were taken using a Leica M80 microscope with a Leica IC80 HD digital camera attached. Image processing and morphological measurements were performed as previously described [25].

### Adenocarcinoma Tumor Model

Swiss nu/nu (7 weeks, *n* = 18) female mice were purchased from Charles River Laboratories-France. Prepubertal mice were ovariectomized under isoflurane anesthesia to remove endogenous ovarian hormone production. Four days before cancer cell injection, mice were implanted s.c. either with a 1.7 mg/60 days MP or with a ME2/60 days RI, or were sham operated (untreated control group). Human adenocarcinoma MCF7 cells were used as previously described [14]. Tumor cells (1 × 10^6^ cells suspended in 200 μl of Matrigel) were injected s.c. to both flanks of mice. Tumors were measured every 2 to 3 days with digital caliper and tumor volume was calculated as V (mm^3^) = *a x b* ^*2*^ *× 0.52* (*n* = 12 tumors per experimental group). At sacrifice, tumors were immediately resected and weighed. The bladder, uterus and kidneys were also dissected, fixed using 4% formalin and embedded in paraffin for histological analysis. Sections were cut at 6 μm, deparaffinised in xylene and rehydrated through graded alcohols, then stained with hematoxylin/eosin. We systematically checked that E2-untreated ovariectomized mice had an atrophied uterus (<10 mg) and that those implanted with an E2-releasing pellet had a significant increase of uterine weight.

### Melanoma Tumor Model

C57BL/6 J (4 weeks, *n* = 18) female mice were purchased from Charles River Laboratories-France. Mice were ovariectomized under isoflurane anesthesia to remove endogenous ovarian hormone production. Two weeks before cancer cell injection, mice were implanted s.c. either with a 0.01 mg/60 days MP or with a ME2/60 days RI, or were sham operated (untreated control group). Mouse melanoma cell line B16K1 (MHC class I–positive B16F10) was used as previously described [16]. Tumor cells (4 × 10^5^ cells suspended in PBS) were injected s.c. to both flanks of C57BL/6 J mice. Tumors were measured every 2 to 3 days with digital caliper and tumor volume was calculated as V (mm^3^) = *a x b* ^*2*^ *× 0.52* and expressed as mean tumor volume (12 tumors per experimental group). At sacrifice, tumors were immediately resected and weighed. We systematically checked that E2-untreated ovariectomized mice had an atrophied uterus (<10 mg) and that those implanted with an E2-releasing pellet had a significant increase of uterine weight.

### Statistical Analysis

All quantitation experiment data are expressed as mean ± SD or mean ± SEM. Data from mammary gland experiments were analyzed by Kruskal-Wallis test and Dunn’s post test. A two-way ANOVA was used for in vivo tumor growth comparisons. Statistical analysis were conducted with GraphPad Prism software. The value of *p* ≤ 0.05 was considered as statistically significant.

## Results

### In Vitro Drug Release Kinetics

**Fig. 1:**
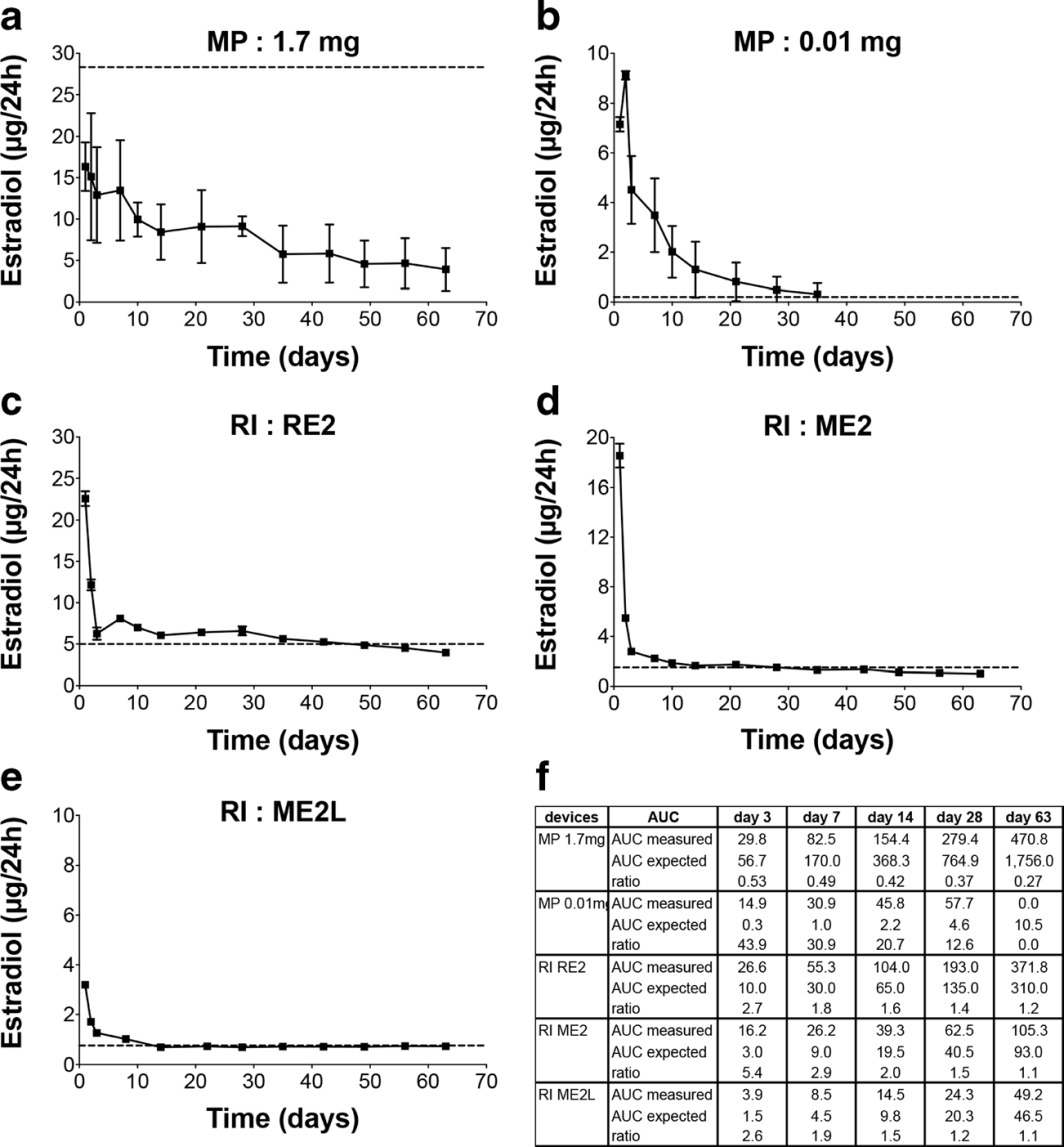
In vitro *E2 release kinetics.* Timeline of in vitro release of E2 MP 1.7 mg/60 days (**a**), MP 0.01 mg/60 days (**b**), RI RE2/60 days (**c**), RI ME2/60 days (**d**) and ME2L/60 days (**e**), over a 63 day period. Dot lines correspond to the expected amount of E2 in accordance with the manufacturer data sheet. Results are expressed in μg/24 h as mean ± SD, *n* = 3. **f** AUC from measured and expected E2 release evaluated at different time points on graphs A to E. AUC ratio corresponds to AUC measured divide by AUC expected

In vitro E2 release kinetic profiles from two MP and three RI slow-releasing devices are illustrated in Fig. 1. Among the available MP, we tested doses of 1.7 mgE2/60 days and of 0.01 mgE2/60 days. Both MP presented a small but long burst effect. For MP 1.7 mg, the mean amount of released E2 was 16.3 ± 2.9 μg/24 h on day 1 (Fig. 1a). Then, it decreased progressively from day 1 to day 35 to reach a stable mean amount of 4.9 ± 0.8 μg/24 h from day 35 to day 63. These released amounts were significantly lower than those announced by the manufacturer (28.33 μg/24 h along 60 days). In accordance, the ratios between measured and expected AUC calculated at different time points along the kinetic were included between 0.53 and 0.27 (Fig. 1f). MP 0.01 mg/60 days was expected to deliver 0.17 μg E2/24 h accordingly to the manufacturer specifications. The mean amounts of E2 released decreased from 9.1 ± 0.2 μg/24 h at day 2 to undetectable level after 35 days while this device was supposed to release for 60 days (Fig. 1b). The mean E2 release between day 3 and day 35 was 1.8 ± 1.6 μg/24 h, an unexpected 10 times higher amount than the expected one. The AUC ratios were far from 1, varying from 43.9 to 0 along the kinetic (Fig. 1f). At each time point, the coefficient of variation (CV) of the E2 mean release by MP was high ranging between 13 and 66%.

All RI devices (RE2, ME2, ME2L) showed similar release profiles (Fig. 1c-e). After a sharp burst effect of 3 days, the RI release kinetics followed a linear steady state period till the end of the tested period (day 63). For RE2 (Fig. 1c), the mean amount of E2 released was 22.6 ± 0.9 μg/24 h at day 1, a 4.5 times higher amount than the 5 μg/24 h given by the manufacturer specifications. It fell to 6.8 ± 1.3 μg/24 h at day 3 and then remained stable with a mean released amount of 5.9 ± 1.2 μg/24 h from day 3 to day 63. For ME2 (Fig. 1d), the mean E2 amount was 18.5 ± 0.9 μg/24 h at day 1, a 12 times higher amount than the 1.5 μg/24 h given by the manufacturer specifications. Then, from day 3 to day 63, the mean release was 1.6 ± 0.5 μg/24 h. For ME2L (Fig. 1e), the mean E2 reached 3.20 ± 0.05 μg/24 h at day 1, a 4.3 times higher amount than the 0.75 μg/24 h given by the manufacturer specifications. At day 3, the mean daily release of E2 fell at 1.26 ± 0.02 μg/24 h to reach a stable amount of 0.71 ± 0.02 μg/24 h from day 14 till day 63. After the burst period of 3 days, these values were consistent with the manufacturer’s specifications. For the three RI devices, the range of AUC ratios at day 3 was 5.4 to 2.6, reflecting the initial burst. Then it varied from 2.9 to 1.1. For all RI devices, the CV of released concentrations remained under 6%.

### In Vivo E2 Plasma Levels

**Fig. 2:**
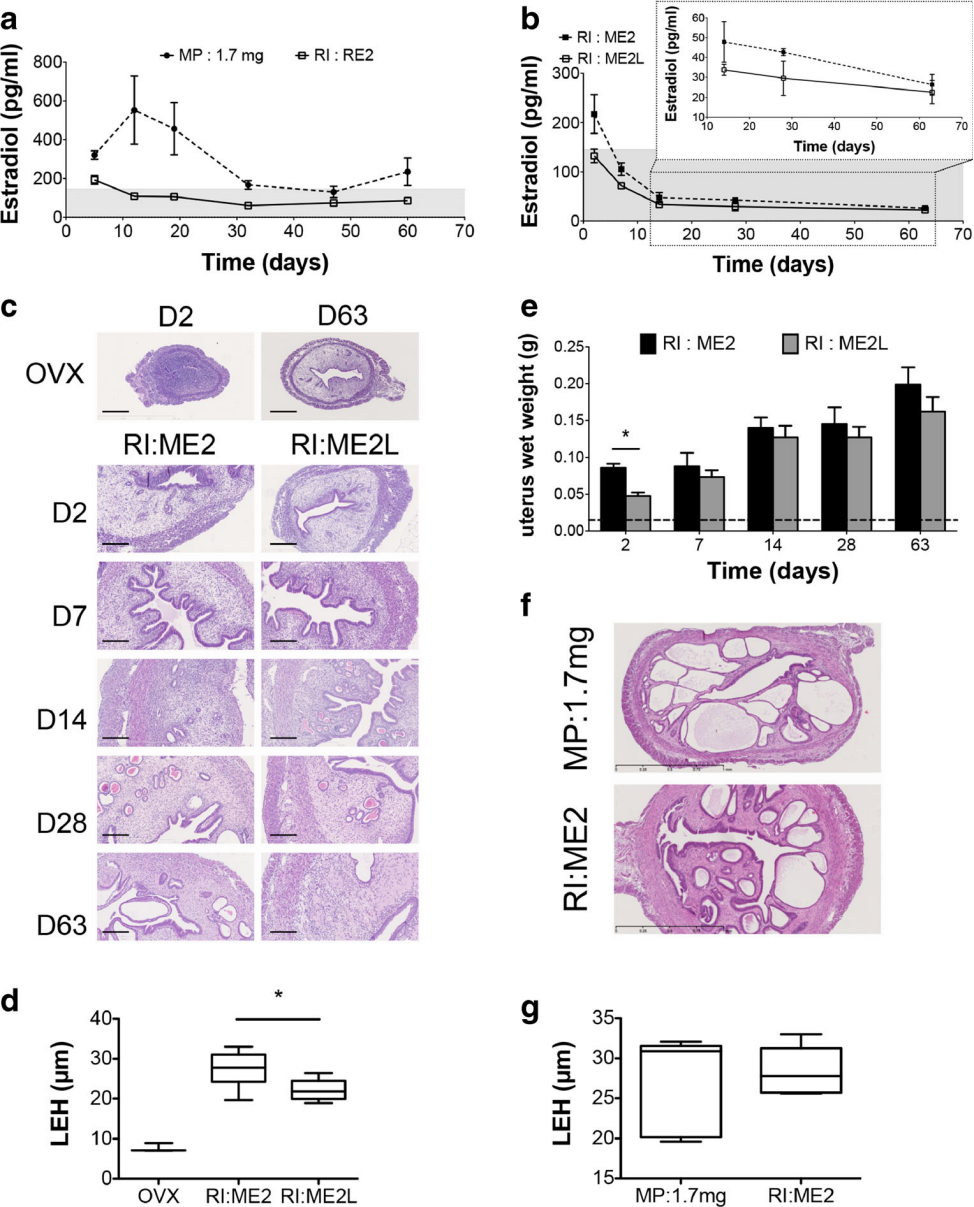
In vivo *E2 release kinetics.* **a** Plasma E2 concentrations measured, over a 63 day period, in plasma of ovariectomized rats following the subcutaneous implantation of MP (1.7 mg/60 days) or RI (RE2/60 days). Results are expressed as mean ± SEM, *n* = 6. **b** Plasma E2 concentrations measured, over a 63 day period, in plasma of ovariectomized mice following the subcutaneous implantation of RI (ME2/60 days or ME2L/60 days). Results are expressed as mean ± SEM, *n* = 4. The grey field represents the physiological E2 range in rodent. Results from day 14 to 63 are zoomed in the small upper graph. **c** Representative histological sections of uterus stained with hematoxylin/eosin, harvested from day 2 (D2) to day 63 (D63) from ovariectomized mice (OVX) or from ovariectomized mice either treated with RI ME2/60 days or with RI ME2L/60 days. Scale bar = 200 μm. **d** Luminal epithelial height (LEH) of uterus after 2 days of treatment, results are expressed in box plots presenting the median and wiskers (min to max), *n* = 4, *p < 0.05. **e** Uterus wet weight of ovariectomized mice following the subcutaneous implantation of RI (ME2/60 days or ME2L/60 days). Dotted line represents the uterus wet weight of OVX mice. Results are expressed as mean ± SEM, *n* = 4, *p < 0.05. **f** Representative histological sections of uterus stained with hematoxylin/eosin, harvested from ovariectomized mice treated either with MP (1.7 mg/60 days) or RI (ME2/60 days) for 8 weeks, scale bar = 1 mm. **g** Luminal epithelial height (LEH) of uterus after 8 weeks of treatment, results are expressed in box plots presenting the median and wiskers (min to max), *n* = 5

The reported physiological range of E2 plasma concentration in rodents is 2.4 to 145 pg/ml [18]. E2 plasma levels obtained in rat over a period of 60 days with MP 1.7 mg/60 days and with RI RE2/60 days are shown in Fig. 2a. Subcutaneously implanted MP 1.7 mg led to supra physiological plasma concentrations with a great variability during the first 3 weeks (mean CV = 50%). It induced a burst reaching a mean E2 plasma concentration of 553 ± 175 pg/ml in plasma after 12 days. Subsequently, the concentrations decreased considerably until day 32 and then remained stable with an average concentration of 177 ± 52 pg/ml that is close to the upper values of the physiological range. E2 plasma concentrations obtained after s.c. RI (RE2) insertion showed a starting burst reaching 193 ± 22 pg/ml after 5 days. Then, after 12 days, E2 plasma concentration was 108 ± 9 pg/ml and was thereafter maintained within the physiological range during the entire experiment (mean CV = 15%). RI ME2 caused an initial burst in mice E2 plasma level of 217 ± 39 pg/ml at day 2 (Fig. 2b). Then it reached physiological concentrations after 7 days (106 ± 13 pg/ml). A steady-state release, giving a mean of 38 ± 11 pg/ml plasma concentration, was observed from day 14 during the entire experiment. Plasma concentrations produced by RI ME2L stood within the physiological range during the entire experiment, starting at 135 ± 13 pg/ml at day 2 to reach a steady-state release of 28 ± 6 pg/ml. Individual variations remained low (within 10%).

Histological sections (Fig. 2c), endometrial luminal epithelial height (LEH) (Fig. 2d) and wet weight (Fig. 2e) of uterus collected revealed a higher impact of RI ME2 than RI ME2L after 2 days of treatment. However, from day 7 to 63 of exposure, the uterine wet weights increased progressively with no significant difference at each time point between both treatments. Uterus treated with MP 1.7 mg or RI ME2 presented the same histological characteristics and luminal epithelial height after 8 weeks of treatment (Fig. 2f-g). However, interindividual variability was higher with MP device.

### Mammary Gland Growth

**Fig. 3:**
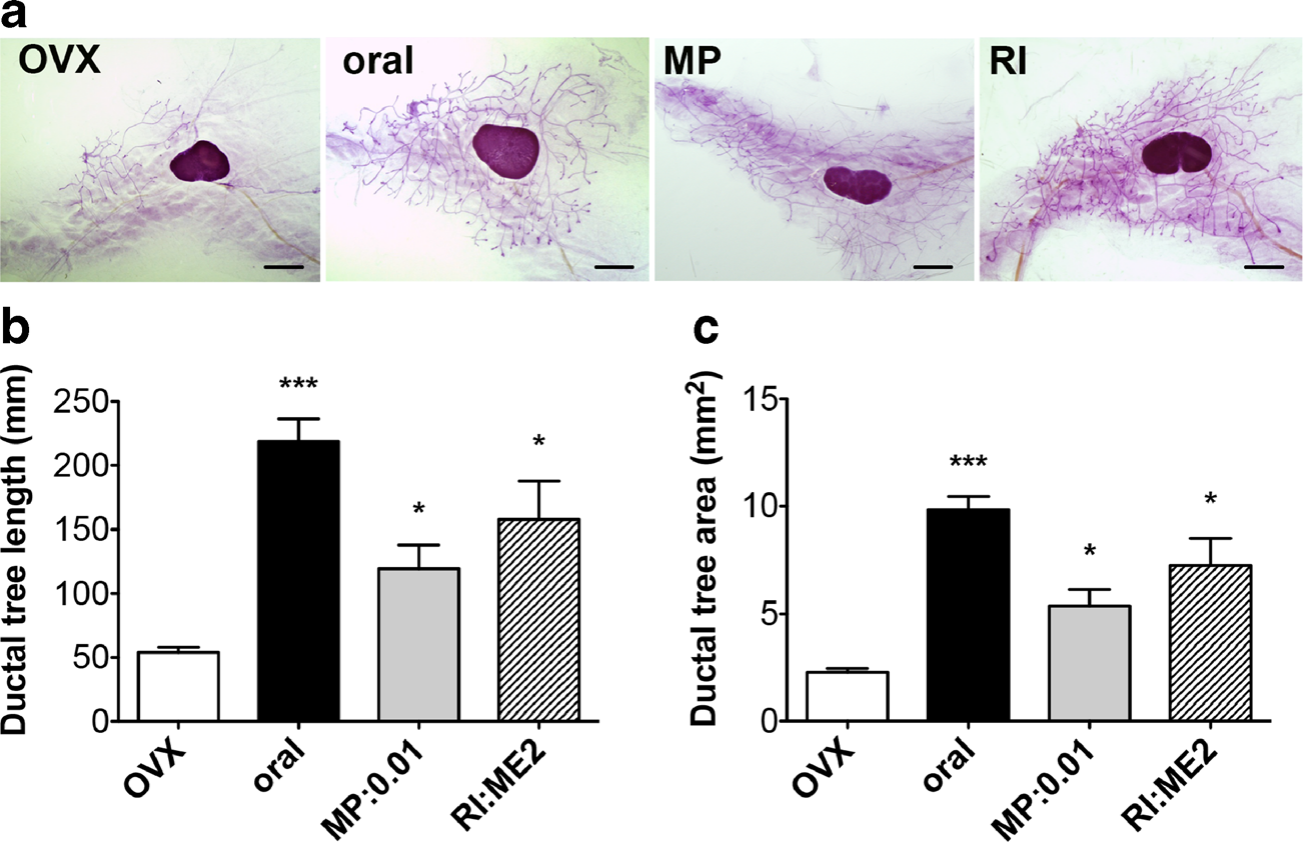
*Mouse mammary gland growth.* **a** Representative photomicrographs of whole mount mouse mammary gland stained with carmin from ovariectomized (OVX) mice or from ovariectomized mice treated with E2 1 mg/kg/day administered by oral gavage (oral), with MP 0.01 mg/60 days (MP) or with RI RE2/60 days (RI), scale bar =1.6 mm. Quantitative analysis of mammary gland development assessed by the automated detection of the ductal tree length (**b**) or of the ductal tree area (**c**). Ovariectomized mice (OVX) were treated or not with E2 1 mg/kg/day administered by oral gavage (oral), with MP 0.01 mg/60 days (MP:0.01) or with RI ME2/60 days (RI:ME2). Results are expressed as mean ± SEM, *n* = 5 per treatment group. *p < 0.05, ***p < 0.001 vs OVX

The in vivo impact of E2-releasing MP and RI devices was analyzed on mammary gland growth of ovariectomized mice and compared to E2 oral administration (Fig. 3a) used as positive control as previously reported [13]. In this previous study, we established that oral administration of 1 mg/kg/day of E2 led to a serum concentration of 171 ± 15 pg/ml (0.63 nM) close to the upper range of physiological concentrations [18]. The mammary gland growth rate was quantified by computer assisted image analysis, by measuring the total length and total area of the epithelial ductal tree as previously described [25]. In the present study, we compared the efficacy of MP 0.01 mg E2 and RI ME2 since both deliver comparable amounts of E2 from 2 to 14 days (see in vitro kinetics Fig. 1b and d). After 14 days of E2 treatment, the average length and area of the epithelial network were significantly increased with both MP (0.01 mg/60 days) and RI (ME2/60 days) devices (Fig. 3b-c). Ductal tree length reached 119 ± 59 mm and 157 ± 67 with MP 0.01 and RI ME2 respectively. Ductal tree area was 5.4 ± 2.4 mm^2^ and 7.3 ± 2.8 mm^2^ with MP 0.01 and RI ME2 respectively. There was no statistical difference between both devices.

### Human MCF7 Adenocarcinoma Xenograft

**Fig. 4:**
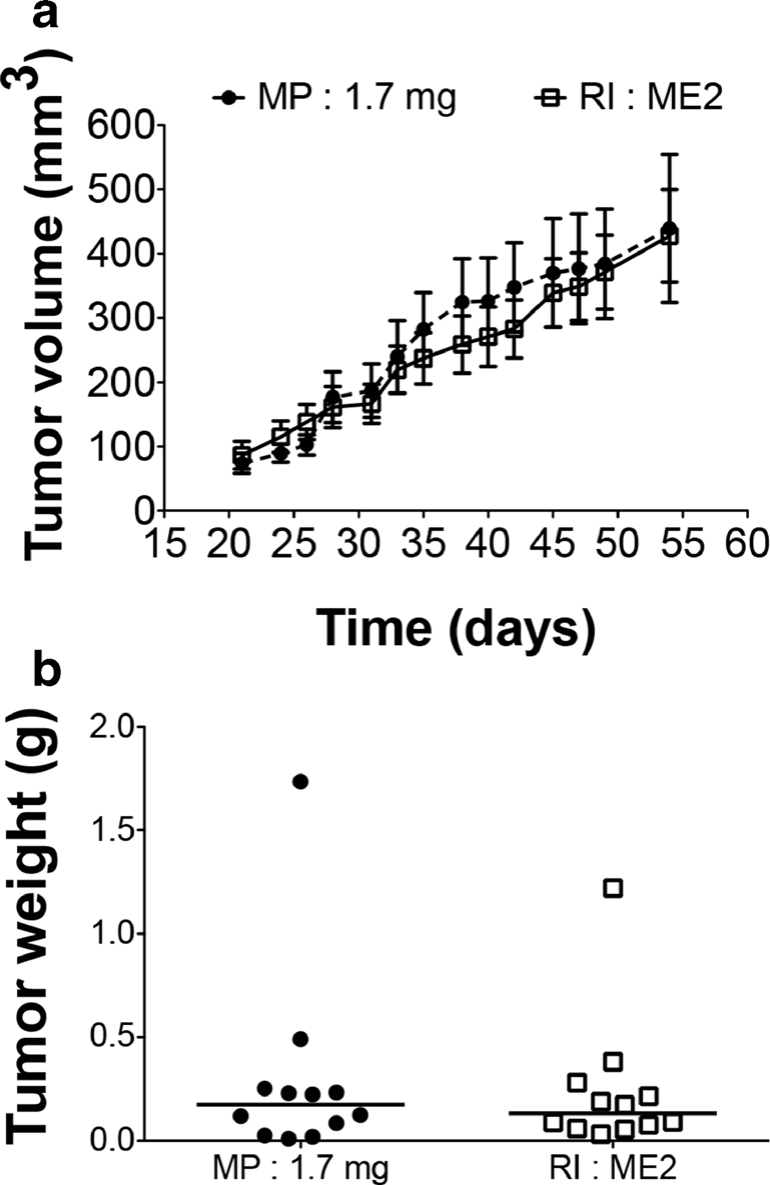
*MCF7 adenocarcinoma xenograft.* **a** In vivo growth curves of human adenocarcinoma MCF7 tumors growing in ovariectomized Swiss nu/nu mice treated with MP 1.7 mg/60 days or with RI ME2/60 days. Results are expressed as mean ± SEM, *n* = 12. **b** Dot graph of tumor weight at sacrifice, median is presented

**Fig. 5:**
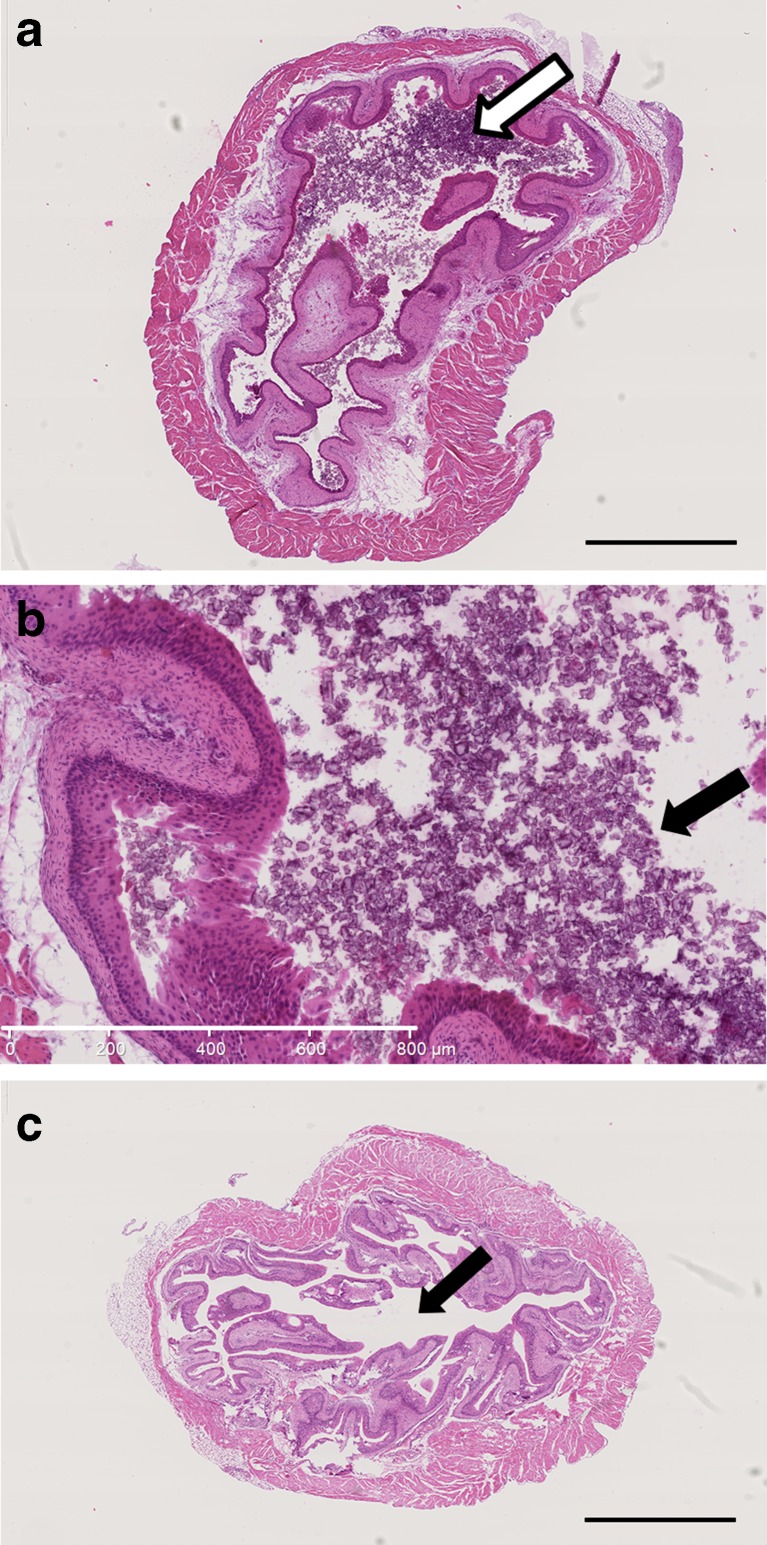
*Bladder stone formation.* **a**, **b** Representative photomicrographs of hematoxylin/eosin staining of bladder from Swiss nu/nu mice supplemented with MP 1.7 mg/60 days for 8 weeks. Stone formation in the bladder lumen is pointed by the arrow. Scale bar = 1.5 mm (**a**), scale bar = 800 μm (**b**). **c** Representative photomicrograph of hematoxylin/eosin staining of bladder from Swiss nu/nu mice supplemented with RI (ME2/60 days). The absence of stone formation in the lumen is highlighted by the arrow, scale bar = 1.5 mm

The xenograft of human MCF7 breast cancer cells is a commonly used in vivo model to study estrogen-dependent breast cancer. The most commonly used and recommended E2-delivering device for this model is the MP 1.7 mg/60 days, which delivers a plasma E2 concentration of 550–900 pg/ml [26, 27] and allows the proliferation of estrogen-dependent tumor cells [6, 14, 28]. In this set of experiment, we compared the efficiency of MP (1.7 mg/60 days) and of RI (ME2/60 days) to promote MCF7 tumor growth in vivo. At the end of the experiment the E2 plasma concentrations were 603 ± 50 pg/ml and 30 ± 7 pg/ml for MP 1.7 mg and RI ME2 treated groups respectively. Both MP and RI devices equally enhanced tumor growth (Fig. 4). Upon sacrifice of MP treated mice, urethral occlusion, leading to urine retention, caused by bladder stone formation was observed in 3 out of 6 mice (Fig. 5a-b). No such complication was observed in any RI treated mice (Fig. 5c).

### Mouse Melanoma Graft

**Fig. 6:**
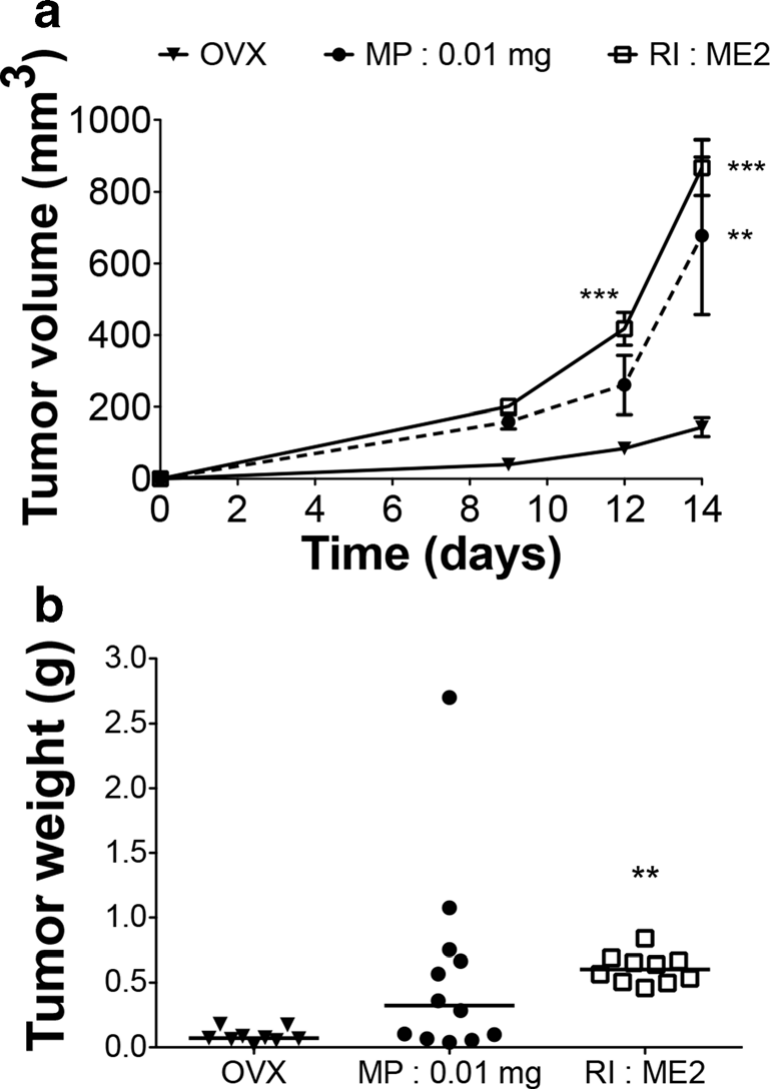
*B16K1 melanoma graft.* **a** In vivo growth curves of melanoma B16K1 tumors injected in ovariectomized mice (OVX) treated with MP (0.01 mg/60 days) or with RI (ME2/60 days). Results are expressed as mean ± SEM, *n* = 9 to 12, **p < 0.01, ***p < 0.001 vs OVX. **b** Dot graph of tumor weight at sacrifice, median is presented, **p < 0.01 vs OVX

The B16K1 melanoma tumor model was previously developed using MP with a low dose of E2 (0.01 mg/60 days) [16]. This MP device was compared to the use of RI ME2/60 days (Fig. 6) that presented a close in vitro pharmacodynamics (see Fig. 1b-d-f). At the end of the experiment the E2 plasma concentrations were 32 ± 22 pg/ml and 35 ± 6 pg/ml for MP 0.01 mg and RI ME2 treated groups respectively. Both devices induced an overall similar increase of tumor growth compared to OVX group (Fig. 6a), even if the data obtained with RI ME2/60 days were statistically more robust. The tumor weights were not significantly increased in animal treated with MP device in comparison with the untreated mice (Fig. 6b). This could be due to large interindividual variations observed both in tumor weight and plasma E2 concentration at the end of the experiment. In animal treated with RI the tumor weights were significantly increased with a low interindividual variation.

### Statistical Power Analysis

A statistical power analysis using the standard deviations was performed on the data obtained with the melanoma experiment on day 14. If an expected mean tumor size of 900 mm^3^ is computed for the treated group, a statistical power of 0.80 is achieved with *n* = 7/group for MP, whereas RI requires only *n* = 3/group to reach a similar statistical power. Conversely, a higher statistical power is achieved with the RI for a given number of subjects per group. For example, with *n* = 5/group, a statistical power of 0.63 is achieved with MP, whereas a statistical very close to 1.00 is obtained with RI.

## Discussion

The use of ready-to-use commercial slow releasing devices to administer steroids, especially estrogens, to small experimental animals remains the method of choice in terms of animal well-being and of safety for both the researcher and the animal. In this study, we evaluated and compared the release kinetic of E2 over sixty days from two different slow-releasing systems MP and RI. We here observed that the real amount of E2 that is released from these devices could differ from manufacturer specifications. The MP we tested did not deliver the expected amount of E2. The RI presented a sharp but short initial burst before reaching the expected delivery amount. The interindividual variability was reduced with RI devices compared to MP. These observations could impact the design, the reliability and the reproducibility of preclinical animal studies, depending on the sensitivity of the physiological or pathological readouts.

Kinetics comparing the release of E2 from MP or RI system revealed, both in vitro and in vivo, the induction of a starting burst effect that could be a potential limitation especially for highly sensitive dose-dependent tissue targets. In vitro kinetics revealed a sharp but short initial burst for RI systems. MP devices presented smaller but longer burst than RI. In vivo, the initial bursts were limited to 1 week with the RI system, although it was observed along at least 3 weeks with the MP. The in vitro kinetics we performed suggest that a 3 day pre-incubation of RI devices in phosphate buffer before s.c. implantation in rodent could prevent this starting burst. However, a pre-incubation of MP systems is not recommended due to the risk of pellet disintegration. The variations between both devices can be explained by differences in the mechanism of diffusion.

Surprisingly, both MP devices were far from delivering the expected amount of E2. For MP 0.01 mg/60 days, the amount of E2 that was released was 44 to 13 times too elevated. It decreased continuously to an undetectable level after 35 days when it was supposed to release during 60 days. In vitro, we showed that the MP 1.7 mg/60 days released only the half of the expected amount of E2. This device was supposed to release a supra physiological dose of 1.1 mg/kg/day in mice and a physiological dose of 0.09 mg/kg/day in rats. The plasma concentrations we measured from rats fluctuated from supra physiologic (± 500 pg/ml) to physiological ones (± 180 pg/ml). These data are in accordance with the observations of other authors that have evaluated kinetics of several E2 MP doses in ovariectomized mice [17] and rats [18, 21, 23, 29]. After day 12–14, the kinetics obtained with the RI systems in rats and mice presented a steady-state level that was maintained along 7 weeks, following a *quasi*-zero order kinetic. This stable kinetic is particularly interesting since the assessment of E2 plasma concentration is most often measured at only one time point from plasma obtained during the sacrifice of the animals. However, if a specific experimentation requires an accurate steady-state release within the 14 days following RI implantation, this initial burst could be a limitation of RI devices. Altogether, these observations have a major impact on the design of experimental protocols, if a specific dose and a stable and reliable treatment is required.

Analysis of uterus histology, luminal epithelial height and wet weight revealed that this tissue is dose-dependent sensitive to E2 under acute short-term administration (2 days in this study). Uterus wet weight increased for all conditions along time exposure, reaching 0.14 to 0.19 g. However, after 1 week of treatment, there was no difference between the various doses and devices administrated (RI ME2, RI ME2L, MP 1.7 mg) in term of wet weight and histological characteristics. These observations are corroborated by the study of Abot et al. [30] who reported that a chronic administration of E2 by MP 0.01 mg induced uterine wet weight of 0.15 g, although it was of 0.02 g after 24 h of treatment with E2 8 μg/kg/day.

To our knowledge, subcutaneous slow-release formulations were not usually used to study the impact of E2 on mammary gland. Subcutaneous injections or oral administrations were more often reported [13, 25, 31]. Based on the specifications provided by the manufacturers, MP 0.01 mg should release 0.17 μg/24 h and RI ME2 1.5 μg/24 h. However, our in vitro release study showed that both devices released comparable amount of E2 from day 2 to day 28. If both devices stimulate ductal tree elongation, RI ME2 has a tendency to produce a higher effect. This could be explained by the acute burst observed with RI ME2 at day 1. Our results clearly show that E2 administered with MP or RI devices could be an interesting alternative for such experiments performed on long-term period. However, attention should be paid to choose a device and a dose that fits the best to a defined experimental design.

In the human adenocarcinoma MCF7 tumor xenograft model, our results clearly show that the supra physiological dose of E2 usually administered with MP [6, 14] are not necessary to allow the proliferation of estrogen-dependent tumor cells. These data corroborates the observations of Dall et al. [26]. Indeed, the RI ME2 was sufficient to induce similar tumor growth kinetic. To note, we observed that despite an initial burst reaching a plasma concentration of 217 pg/ml at day 2, this device released physiological concentrations of E2 from day 7. This is of great interest since several studies [20, 32–34] reported that high doses of E2, administered for long-term period, are associated with renal damage, urethral occlusion and bladder stone formation, corroborating our observations with E2 MP 1.7 mg/60 days used along a period of 8 weeks. The use of RI (ME2/60 days) releasing long-term physiological dose of E2 appears more appropriate since no such complication was observed in mice bearing these implants. Kang et al. [32] showed that mortality rate was significantly reduced when mice received a lower but nonetheless active dose of E2. Nevertheless, we do not exclude that this side effect could vary with mouse strain and treatment duration. It should also be evaluated for the development of patient-derived xenograft (PDX) model requiring very long-term supplementation of E2.

The comparison of MP and RI presenting close in vitro pharmacodynamics revealed overall similar impact on B16K1 melanoma tumor growth. The differences observed between both devices regarding the amplitude of the initial burst of 2 days have no major impact on this readout. However, standard errors and dispersion of tumor volume and weight were significantly lower in RI treated group than in MP one. This is in accordance with the standard deviations and the coefficient of variation we measured in the in vitro and in vivo release kinetics, as well as in plasma levels obtained at the end of the experiment. Some disintegrated matrix pellets were also found after sacrifice. This could contribute to the larger variability in the MP group. The RI presents reduced intra-group variability that could result from a better control of release mechanisms as shown by in vitro pharmacodynamics. Interestingly, our statistical power analysis revealed that the use of RI could reduce the number of animal necessary to reach sufficient statistical power in specific experiments. This is of particular interest to achieve the “reduction” criteria of the 3Rs rule (Replacement, Reduction, Refinement) [35, 36]. These guidelines governing animal ethic are explicitly required by EU legislation (http://eur-lex.europa.eu/legal-content/EN/TXT/?qid=1468335661396&uri=CELEX:32010L0063). Moreover, reduction of animal number to minimum is a major concern for scientific working with experiment animals not only for ethic, but also for scientific, legal and economic purposes.

In conclusion, this work highlights that drug release mechanisms, MP or RI, used to deliver E2 could impact the amount of E2 that is really deliver and the interindividual variability. Depending on the dose-dependent sensitivity of the physiological or pathological readout studied, careful attention should be paid to choose the slow-releasing device that is the most appropriated to a specific experiment. In the context of ever increasing legal requirements for the welfare and protection of animals used for scientific purposes, the judicious choice of a reliable and accurate drug release system will fulfill the 3Rs rule.
